# A randomized controlled efficacy trial of an mHealth HIV prevention intervention for sexual minority young men: MyPEEPS mobile study protocol

**DOI:** 10.1186/s12889-020-8180-4

**Published:** 2020-01-15

**Authors:** Lisa M. Kuhns, Robert Garofalo, Marco Hidalgo, Sabina Hirshfield, Cynthia Pearson, Josh Bruce, D. Scott Batey, Asa Radix, Uri Belkind, Haomiao Jia, Rebecca Schnall

**Affiliations:** 1 Division of Adolescent Medicine, Ann & Robert H. Lurie Children’s Hospital of Chicago, 225 E. Chicago Avenue, Box 161, Chicago, IL 60611 USA; 2 Department of Pediatrics, Feinberg School of Medicine, Northwestern University, Chicago, IL USA; 3 Children’s Hospital of LA, Los Angeles, CA USA; 4 Department of Medicine, SUNY Downstate Health Sciences University, Brooklyn, NY USA; 5 University of Washington, Seattle, WA USA; 6 Birmingham AIDS Outreach, Birmingham, AL USA; 7 University of Alabama at Birmingham, Birmingham, AL USA; 8 Callen-Lorde Community Health Center,, New York, NY USA; 9 Columbia University Mailman School of Public Health, New York, NY USA; 10 Columbia University, School of Nursing, New York, NY USA

**Keywords:** HIV prevention, Young men, Sexual minority, Intervention

## Abstract

**Background:**

Young sexual minority men in the United States have a high incidence rate of HIV infection. Early intervention among this group, that is timed to precede or coincide with sexual initiation, is of critical importance to prevent HIV infection. Despite this, there are very few published randomized controlled efficacy trials testing interventions to reduce sexual vulnerability for HIV acquisition among racially/ethnically diverse, very young, sexual minority men (aged ≤18 years). This paper describes the design of a mobile app-based intervention trial to reduce sexual risk for HIV acquisition and promote health protection in this group.

**Methods:**

This study is a randomized controlled trial of an mHealth-based HIV prevention intervention, MyPEEPS Mobile, among diverse sexual minority cisgender young men, aged 13–18 years. The mobile intervention was adapted from a prior group-based intervention curriculum with evidence of efficacy, designed to be specific to the risk contexts and realities of young sexual minority men, and to include psychoeducational and skill-building components with interactive games and activities. Participants are recruited locally within four regional hubs (Birmingham, AL, Chicago, IL, New York City, NY, Seattle, WA) and nationwide via the Internet, enrolled in-person or remotely (via videoconference), and randomized (1:1) to either the MyPEEPS Mobile intervention or delayed intervention condition. Post-hoc stratification by age, race/ethnicity, and urban/suburban vs. rural statuses is used to ensure diversity in the sample. The primary outcomes are number of male anal sex partners and frequency of sexual acts with male partners (with and without condoms), sex under the influence of substances, and uptake of pre-and post-exposure prophylaxis, as well as testing for HIV and other sexually transmitted infections at 3-, 6- and 9-month follow-up.

**Discussion:**

Behavioral interventions for very young sexual minority men are needed to prevent sexual risk early in their sexual development and maturation. This study will provide evidence to determine feasibility and efficacy of a mobile app-based HIV prevention intervention to reduce sexual risk among this very young group.

**Trial registration:**

ClinicalTrials.gov number, NCT03167606, registered May 30, 2017.

## Background

Young men who have sex (YMSM) with men are vulnerability to HIV infection, particularly racial/ethnic minority YMSM. In the United States in 2017, YMSM made up 93% of all new cases of HIV infection among youth age 13–24 years, with YMSM of color (Black or Latinx) comprising the vast majority of those cases (76%) [1]. Psychosocial factors, such as bullying and other forms of violence and related feelings of isolation; contextual factors (e.g., family, peer and partner relationships); as well as high number of partners, low rates of condom use, and low rates of testing for HIV and other sexually transmitted infections (STIs), are contributing factors [2]. Intervention among very young sexual minority men, prior to or at the time of sexual initiation, is an important strategy to increase sexual health education and skill-building; indeed, NIH’s *Strategic Plan for HIV* highlights the importance of addressing HIV prevention with key populations at-risk for HIV, including youth as young as 13 [3]. Currently, however, there are no interventions in the Center for Disease Control and Prevention’s Compendium of Evidence-based Interventions that target diverse sexual minority men 18 years of age and younger [4].

Evidence suggests that mHealth-based intervention approaches may be particularly salient for technology savvy youth, but also a promising method to increase reach to key populations with educational information, digital media, and/or game-based learning, aimed at reducing HIV risk behaviors [5, 6]. In a review of mHealth interventions for high risk MSM, Schnall and colleagues found that web-based videos and education modules reduced HIV risk behavior and promoted HIV testing [7]. Among youth ages 13–29, evidence suggests that web-based interactive and educational approaches are efficacious for delaying sexual initiation [8, 9], increasing knowledge of HIV/STIs, and promoting condom self-efficacy [10]. MyPEEPS Mobile is an intervention adapted from a group-based HIV prevention curriculum, developed via formative research [11–13], for diverse YMSM, ages 16–20. The group-based intervention demonstrated evidence of efficacy to reduce sexual risk behavior in this population [14]. We adapted the group-based intervention curriculum to mobile app for a younger (≤18 years of age) and more diverse group (i.e., to include Native Americans, Asian Americans) through a user-centered and iterative design process and tested for feasibility, acceptability, and usability in a recent set of studies [15–18].

### Study objective

The primary objective of this study is to test the efficacy of the MyPEEPS Mobile intervention to reduce sexual risk for HIV acquisition and promote health behavior among young sexual minority men, aged 13 to 18 years.

## Methods/design

### Design

**Fig. 1:**
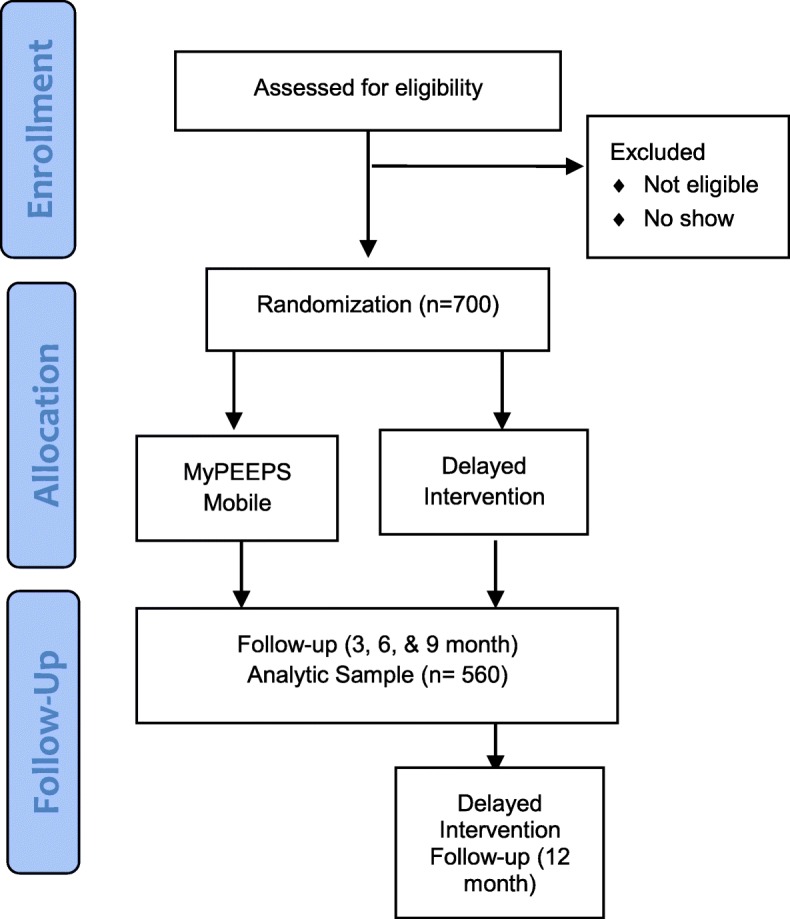
MyPEEPS CONSORT Diagram

This study is a nationwide two-arm randomized controlled trial among racially and ethnically diverse sexual minority young men, aged 13–18 years. Participants are randomized to either the MyPEEPS Mobile intervention or a delayed intervention comparison condition and followed at 3-, 6-, and 9-months post-baseline. The intervention is delivered in the period between baseline and the 3-month follow-up visit. The comparison group is crossed-over to intervention at the 9-month visit and followed to 12-months post-baseline (Fig. 1).

### Identification and recruitment of participants

Participants are recruited locally within four regional hubs (Birmingham, AL, Chicago, IL, New York City, NY, Seattle, WA) and nationwide via the Internet. Local outreach occurs actively in youth- and sexual minority-focused community organizations and events, as well as passively via posted flyers. Internet-based outreach occurs primarily via paid and targeted ads on platforms frequented by adolescents (e.g., Instagram, Snapchat). Eligibility criteria include: (1) ages 13–18 years; (2) assigned male sex at birth and, at the time of enrollment, self-identify as male; (3) understand and read English; (4) live in the United States; and (5) own a smartphone (6) report sexual interest in other men (7) has either kissed another man or plan on having sex with a man in the next year and (7) self-reported HIV-negative or unknown HIV status. Participants are excluded if investigators determine that participation may be detrimental to them or the study (e.g., severe cognitive deficit) or they participated in the pilot phase of the study.

Our goal is to enroll 700 participants overall, and at least 70 participants of each year of age (i.e., aged 13, aged 14, etc.) in each of six racial/ethnic groups [i.e., American Indian or Alaskan native, Asian or Asian American, Black or African American, Latinx (of any race), Native Hawaiian or other Pacific Islander, and White]; and among youth living in rural-designated areas.

### Randomization

On a rolling basis, participants are randomized in blocks of four with a 1:1 ratio to either MyPEEPS Mobile or a delayed intervention condition. The random assignments are generated by a computerized random number generator by the Principal Investigator (RS). Random assignments are concealed from both participants and study staff until they are revealed at the point of randomization after baseline data collection has been completed. The statistician will be blinded to condition for analysis to reduce potential bias during statistical analyses.

### Description of the intervention: MyPEEPS Mobile

MyPEEPS Mobile is based on the Social-Personal Framework [19], which builds on Social Learning Theory [20] by adding important psychosocial (e.g., affect dysregulation) and contextual risk factors (e.g., family, peer, and partner relationships) related to youth vulnerability to HIV risk. MyPEEPS Mobile provides educational information about HIV and STIs among YMSM, raises awareness about minority stress (e.g., due to sexual identity), and builds skills for condom use, emotion regulation, and negotiating interpersonal and substance-related risk. The learning process is facilitated through the stories of four “peeps” (Philip, Nico, Artemio, and Tommy), who are composites of YMSM who participated in the formative phase of the original MyPEEPS intervention development process [11, 12]. A running theme throughout the intervention is sexual risk reduction and goal-setting through an activity called, “BottomLine,” in which participants are challenged to articulate how much risk they are willing to accept for different sexual acts (e.g., anal sex, oral sex) and to continually re-consider these limits after exposure to the intervention activities (i.e., building knowledge, self-awareness, as well as self-efficacy). Using a responsive web design, the conventional web site is viewable on small screens and usable with touch screens. The content is delivered through a series of games, scenarios and role-plays within 21 mobile activities that are divided into four sequential modules or “PEEPScapades,” which are targeted to younger and less sexually experienced sexual minority young men [18]. All content is accessible for the period between randomization and the 3-month follow-up visit (i.e., content does not expire and can be re-visited), and must be completed in a linear manner. Movement through the app is encouraged with both in-app “trophies” and monetary incentives for the time it takes to complete the activities. Privacy is protected via log-in and password credentials and automatic log-off of the app after 20 min of inactivity.

### Delayed intervention condition

Participants in the delayed intervention condition are provided with access to the MyPEEPS Mobile app at the 9-month visit with log-in and password credentials. Procedures for app access and incentives for completion are the same as for the intervention condition. Access is provided through the 12-month study visit.

### Study assessments

Participants are enrolled in-person or remotely (via videoconference with written electronic assent or consent) by study staff, then complete study assessments at baseline and follow-up via computer-assisted self-interviewing (CASI; either in-person or remotely via a web link). Follow-up assessments are conducted at 3-, 6- and 9-month follow-up visits, with an additional follow-up visit at 12-months for the delayed intervention group. Participants are required to show their ID, and the face on their ID are matched to their face on the videoconferencing screen. Each participant was given a survey link matched to their study ID upon confirmation of their identity. All study data are securely stored at the primary study site in a limited access database by study ID. All hard copy participant information (e.g., study checklists, consent forms) are securely stored at each study site in locked file cabinets with limited access.

#### Primary outcome

The primary outcome is number of male sex partners and frequency of anal sex acts with male partners (with and without condoms), sex under the influence of substances, and uptake of pre-and post-exposure prophylaxis (PrEP, PEP), as well as testing for HIV and other STIs (e.g., chlamydia, gonorrhea, hepatitis, HPV/genital warts, syphilis). Items assessing sexual behavior are self-administered and adapted for sexual minority men from the AIDS Risk Behavior Assessment (ARBA) [21]. Items assess sexual behavior in the prior 30 days and 3 months. The basis for construction of the primary outcome is a set of sequential questions asking the participant to estimate the number of sex partners they had in the recall period and the number of condomless sex acts by type of sex (anal, oral, vaginal) with these partners. Items to assess uptake of PrEP, PEP, and HIV testing history are based on those used in prior studies of YMSM [22, 23].

### Statistical analysis

All multivariate analyses will be preceded by standard bivariate analyses to describe key variables and relationships among them. These analyses will include means, frequency tables, histograms, and examination of distributions to promote data quality. All statistical tests will be two-sided tests with the level of significance at 0.05. We propose to use generalized linear mixed models (GLMM) to analyze both count and binary outcomes to determine efficacy of the intervention. For the missing values at the baseline or partial baseline collected data, we will use a multiple imputation (MI) approach [24]. Models will also be run on the non-imputed data with full-information maximum likelihood (FIML) estimation as an alternative for the MI method [25]. Rates of reduction will be calculated controlling for all other covariates in the multivariable model. Models will be calculated by using the GLIMMIX and MIANALYZE procedures in SAS, version 9.4, and model fit will be evaluated by diagnostic statistics and residual plots.

### Sample size calculation

We target enrollment of 700 participants overall, and at least 70 participants of each subgroups of age, racial/ethnicity, and rural-designated areas. We estimated the statistical power for the main outcome (recent number of condomless anal sex acts with male partners) based on two scenarios: (1) to examine overall effect with total subjects; and (2) to conduct stratified analysis to examine the effects in some subgroups (age, racial/ethnicity, and in rural areas). The following assumptions are used for the power estimation: (1) an 80% retention rate (analytic sample = 560 for total, and 56 for subgroups); (2) a conservative and high intra-cluster correlation (ICC) of 0.8, (3) mean number of recent condomless anal sex acts with male partners at baseline is 1.2 [14] and (4) all power estimations are based on α = 0.05 and 2-sided tests. Findings from the prior MyPEEPS study indicate that the post-intervention number of condomless anal sex acts decreased by 63%, or a relative risk (RR) of 0.37 [14]. However, the large effect was not statistically significant. Because the estimated effect size of the intervention was unreliable, instead, we use RR = 0.73, one standard error over the estimated RR of 0.37. This provides a conservative estimation of the minimum sample size need. For the subgroup analyses, we use the effect size of RR = 0.37. To examine overall effect with all participants, we will have 97% power to detect a relative risk of 0.73 with analytic sample size of 560. Secondly, for the stratified analyses, we will have 92% power to detect a relative risk of 0.37 in subgroups with analytic sample size of 56.

Primary study data will be analyzed as soon as possible after the end of data collection, with study findings disseminated in peer-review public health journals.

## Discussion

We describe herein the design of the MyPEEPS Mobile study, a randomized controlled efficacy trial of an mHealth intervention with educational and gaming components to reduce HIV risk behavior and promote protective health behaviors in young sexual minority men aged 13–18. The intervention is based on a prior group-based intervention with evidence of efficacy [14], as well as both theory and empirical evidence for the challenges faced by sexual minority men to protect their own sexual health [11, 13, 22, 26–29]. The design of this study has several strengths, including its focus on very young sexual minority men, prior to or at the point of sexual initiation; testing a mobile-adapted evidence-based intervention informed by the experiences of YMSM; and the rigorous evaluation design, sample size and analysis by age, race/ethnicity, and rural-based subgroups.

The defining characteristics of this intervention are that it focuses on important psychosocial (e.g., affect dysregulation) and contextual factors (e.g., family, peer and partner relationships) related to youth vulnerability to HIV risk. It provides educational information about HIV and STIs among YMSM, raises awareness about minority stress, and build skills for condom use, emotion regulation, and negotiating interpersonal and substance-related risk. Given the vulnerability of young sexual minority men to HIV infection and the current plateau in progress in HIV prevention [30], sexuality education that is specific to their needs and builds skills to manage their own sexual health is of critical importance and is consistent with current recommendations for an empowerment approach to sexuality education [31]. Empowerment education, critical thinking, and communication skill-building provide the foundation for protective sexual health decision-making.

Early intervention is critical, yet there are no evidence-based interventions developed specifically for very young sexual minority men. Their vulnerability increases dramatically during the period of adolescence and young adulthood. In 2017, less than 1% of youth who received an HIV diagnosis were aged 13 to 14, 21% were aged 15 to 19, and 79% were aged 20 to 24 [1]. This study provides important information and skills prior to or coincident with sexual initiation, to inform sexual decision-making and directly address this developmental trajectory of vulnerability. Furthermore, MyPEEPS Mobile is delivered in a mobile app-format aimed at increasing both uptake and scalability. Our web-based outreach approach and mobile app format increase the potential reach of this intervention to this young group.

Finally, this intervention is well powered to detect effects in a randomized trial design, which increases scientific rigor. In addition, it is focused on gathering a diverse sample by age, race/ethnicity, and rural representation in sufficient size for testing of subgroup effects, which increases generalizability. If this trial of MyPEEPS Mobile demonstrates evidence of efficacy, this approach has potential for broad public health impact.

## Data Availability

Not applicable.

## Abbreviations

ARBA: AIDS Risk Behavior Assessment
CASI: Computer Assisted Self Interviewing
FIML: Full Information Maximum Likelihood Estimation
GLMM: Generalized Linear Mixed Models
HIV: Human Immunodeficiency Virus
ICC: Intra-Cluster Correlation
mHealth: Mobile Health
MI: Multiple Imputation
PEP: Post-Exposure Prophylaxis
PrEP: Pre-Exposure Prophylaxis
RR: Relative Risk
STI: Sexually Transmitted Infection
YMSM: Young Men Who Have Sex with Men
